# Alterations in Nerve-Evoked Bladder Contractions in a Coronavirus-Induced Mouse Model of Multiple Sclerosis

**DOI:** 10.1371/journal.pone.0109314

**Published:** 2014-10-13

**Authors:** Neil S. Lamarre, Alan S. Braverman, Anna P. Malykhina, Mary F. Barbe, Michael R. Ruggieri

**Affiliations:** 1 Department of Anatomy and Cell Biology, Temple University School of Medicine, Philadelphia, Pennsylvania, United States of America; 2 Division of Urology, Department of Surgery, Perelman School of Medicine, University of Pennsylvania, Glenolden, Pennsylvania, United States of America; Max-Delbrück Center for Molecular Medicine (MDC), Germany

## Abstract

**Background:**

Patients with neurodegenerative diseases such as multiple sclerosis, Parkinson’s, and Alzheimer’s often present with lower urinary tract symptoms (LUTS, urinary frequency, urgency, nocturia and retention) resulting from damage to the peripheral and central nervous systems. These studies were designed to examine the changes in the function of the bladder that may underlie neurogenic bladder dysfunction using a mouse model of demyelination in the CNS.

**Methods:**

Bladders from 12 week old male C57BL/6J mice with coronavirus-induced encephalomyelitis (CIE, a chronic, progressive demyelinating disease model of human MS), and age-matched controls, were cut into 5–7 strips and suspended in physiological muscle baths for tension measurement in response to agonists and electric field stimulation (EFS). Experiments were performed on intact and denuded (with mucosa removed) bladder strips.

**Results:**

The maximum effect of EFS was not significantly different between CIE and control bladders. Nerve-evoked EFS contractions (tetrodotoxin-sensitive) were blocked by a combination of atropine (cholinergic antagonist) and α,β-methylene ATP (an ATP analog that desensitizes purinergic receptors). In response to EFS, the α,β-methylene ATP-resistant (cholinergic) component of contraction was significantly reduced, while the atropine-resistant (purinergic) component was significantly increased in CIE bladders. Removal of the mucosa in CIE bladders restored the cholinergic component. Bethanechol (muscarinic receptor agonist) potency was significantly increased in CIE bladders.

**Conclusions:**

Our data demonstrate a deficit in the nerve-evoked cholinergic component of contraction that is not due to the ability of the smooth muscle to respond to acetylcholine. We conclude that neurodegenerative bladder dysfunction in this model of multiple sclerosis may be due, in part, to pathologic changes in the mucosa that causes suppression of muscarinic receptor-mediated contractile response and augmentation of purinergic response of the underlying muscle. Further studies utilizing CIE mice should help elucidate the pathological changes in the mucosa resulting from demyelination in the CNS.

## Introduction

Multiple sclerosis (MS) is a chronic autoimmune disorder, in which the improper activation of the host’s immune system results in demyelinated lesions, predominantly in the brain and spinal cord. Typically diagnosed in young adults, the progressive neurodegeneration can have a dramatic impact on their quality of life. As the disease progresses, the prevalence and severity of LUTS often parallel the disease progression [1]. A collection of symptoms including urgency and frequency of micturition, incontinence, incomplete emptying, hesitancy, weak urine stream and urinary retention are common in MS patients, as well as other patients with neurogenic bladder [2], [3]. Neuronal control over bladder function is coordinated by the pontine storage and micturition centers in the brain stem, which sends projections to regions of the lumbosacral spinal cord coordinating parasympathetic outflow to the detrusor. It is thought that the hyperreflexia observed in neurogenic bladder dysfunction may be a result of impaired neuronal connections between these areas of the spinal cord and the pontine micturition center [4].

Human MS is a multifactorial disease, with a role for both genetic and environmental influence. One such environmental variable that may trigger an immune response to self-antigens is viral infection. Virus-induced models of demyelinating disease are available [5], [6], but rarely used to study neurogenic bladder dysfunction. Experimental autoimmune encephalomyelitis (EAE) is a rodent model of MS that is used more frequently to study neurogenic bladder dysfunction. It is initiated by injections with myelin basic protein, or spinal cord homogenates, with adjuvants to initiate the immune response. Animals with EAE show micturition symptoms common to MS patients, including detrusor hyperactivity [7], [8], shortened intermicturition intervals, decreased voiding volumes, morphology changes and fibrosis in the bladder [9], [10]. However, while EAE produces chronic inflammation in the CNS, it has an unclear onset and limited demyelination and neurodegeneration [11].

Currently, therapeutic options are not specific for neurogenic bladder, lack efficacy, and have poor patient adherence [12]. Antimuscarinic drugs are the first-line of treatment, however there is little evidence to support their efficacy in neurogenic bladder dysfunction [12], [13]. A better understanding of the pathological changes in bladder innervation and effects of nerve degeneration on the contractile mechanisms of the detrusor will lead to the development of much-needed treatment options for this subset of patients. The aim of this study was to examine the nerve-evoked, cholinergic and purinergic contractions of the bladder *in* *vitro* using a coronavirus-induced mouse model of MS.

## Materials and Methods

### Materials

All drugs and chemicals were obtained from Sigma Chemical Company (St. Louis, MO) except for darifenacin (which was a generous gift from Pfizer Limited, Sandwich, Kent).

### Ethics Statement

CIE induction and clinical symptom score assessment were approved by the University of Pennsylvania Institutional Animal Care and Use Committee, under protocol #816570 (approved December 2012). Bladder contraction experiments in these mice, and age-matched controls were approved by Temple University Institutional Animal Care and Use Committee (protocol #4240, approved August 2013). Euthanization of animals via cervical dislocation was performed following isoflurane anesthesia, in accordance with the recommendations in the Guide for the Care and Use of Laboratory Animals distributed by the National Institutes of Health.

### Mouse model of coronavirus-induced encephalomyelitis (CIE)

**Figure 1:**
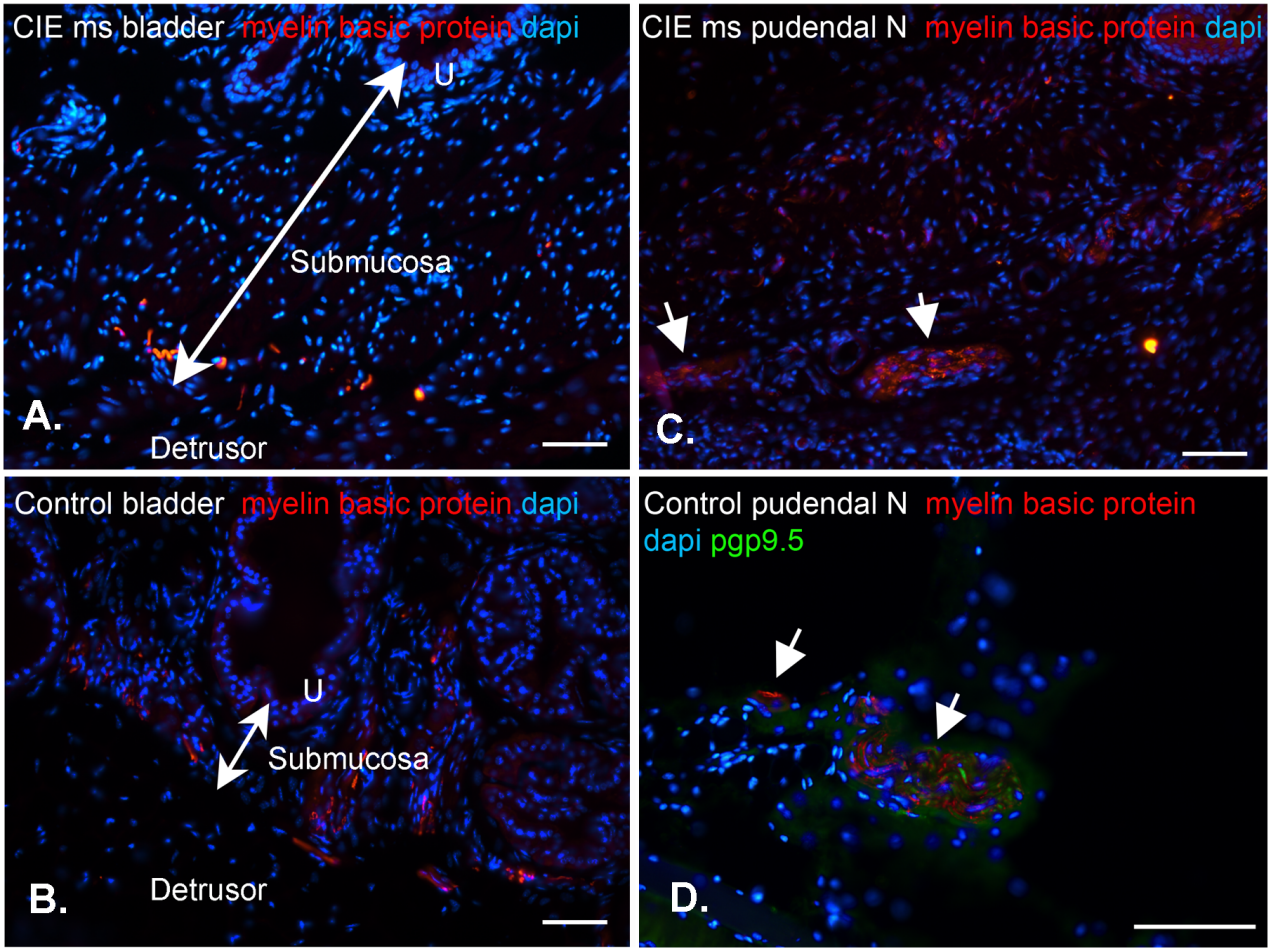
Immunostaining for myelin basic protein (MBP; red), dapi (blue) nuclear marker, and pgp9.5 (green) axonal marker. MBP positive staining in CIE mouse bladder wall (A) and pudendal nerve (C) was similar to age matched control mouse bladder wall (B) and pudendal nerve (D). Thus, there is no evidence of peripheral demyelination in CIE mice compared with age matched control (D) for reference. Scale bars = 50 µm.

Male mice (C57BL/6J, 8 wks of age, N = 54, Jackson Laboratories, Bar Harbor, ME) were injected with mouse hepatitis virus (MHV, A59 strain, 5,000 PFU) in 20 µl of phosphate buffered saline (PBS) intracranially. A Clinical Symptom Score (CSS) was determined for each animal following induction of CIE based on the following observations: 0, normal with no clinical signs; 1, loss of tail tonicity/kyphosis; 2, tail paralysis/severe kyphosis; 3, partial hind limb paralysis; 4, complete hind limb paralysis; 5, complete hind limb paralysis and forelimb paresis/paralysis. In this model, most animals show significant CSS scores within a few days and the CSS score peaks 2 weeks post-inoculation and then declines [14], [15]. Thus, CSS scores were determined for each animal until the CSS score declined for 3 days, usually during the second week. The average maximum CSS was 2.0±0.12 for the 54 animals used in this study. The majority of animals with a CSS of 4–5 do not survive the acute phase of encephalitis (two animals in this study survived with a peak CSS of 5 during acute infection, none with a CSS of 4). Demyelinated lesions in the CNS are apparent at this time point, as previously established with this model [16], [17], however we observed no demyelination in the bladder wall at week 4 post-infection (Figure 1). All mice had ad libitum access to food and water.

Mice were euthanized 4 weeks following the induction of CIE and the bladders were harvested. The urothelium and submucosa were removed from some bladders (mucosa denuded) by sharp dissection with the use of a surgical microscope. The bladders were cut into longitudinal strips approximately 2 mm wide and 5–6 mm long. The bladder strips were then suspended with 0.75 g of tension in tissue baths containing 10 ml of modified Tyrode’s solution (125 mM NaCl, 2.7 mM KCl, 0.4 mM NaH_2_PO_4_, 1.8 mM CaCl_2_, 0.5 mM MgCl_2_, 23.8 mM NaHCO_3_, and 5.6 mM glucose) and equilibrated with 95/5% O_2_/CO_2_ at 37°C.

Following equilibration to the bath solution for 30 minutes, the strips were then induced to contract with high potassium Tyrode’s solution (7.7 mM NaCl, 120 mM KCl, 0.4 mM NaH_2_PO_4_, 1.8 mM CaCl_2_, 0.5 mM MgCl_2_, 23.8 mM NaHCO_3_, and 5.6 mM glucose) then rinsed. Data was collected on a computer using a PowerLab data acquisition system (ADInstruments, Inc. Colorado Springs, CO).

### Bethanechol concentration response curves

The strips were incubated for 30 minutes in the presence or absence of one of 3 concentrations of the competitive M_2_ selective antagonist methoctramine (0.1, 10, or 100 µM, n = 4–6 per condition) or the competitive M_3_ selective antagonist darifenacin (1, 3, or 10 nM, n = 4–6 per condition). Dose response curves were derived from the peak tension developed in response to cumulative addition of the non-subtype selective muscarinic receptor agonist bethanechol. Bethanechol concentrations from 0.1 µM up to 10 mM (at half log intervals) were used with approximately 3 minutes intervals before the next concentration of bethanechol. Either vehicle or one concentration of methoctramine (M_2_ selective antagonist) or darifenacin (M_3_ selective antagonist) was used for each muscle strip. Dose ratios for antagonist treated strips were determined based on the average EC_50_ of the vehicle (H_2_O) treated strips. EC_50_ values were determined for each strip using a sigmoidal curve fit of the data (Origin, Originlab Corp. Northampton, MA.). Schild plots were constructed to calculate antagonist potency.

### α,β-methylene ATP concentration response curves

Because the purinergic (P2X) receptors involved in bladder contraction are known to desensitize rapidly, each bladder strip was exposed to only 1 concentration of α,β-methylene ATP (0.3 to 300 µM at half log intervals, n = 4–9) to generate α,β-methylene ATP concentration response curves.

### Nerve-evoked contractions

Nerve-evoked contractions were induced by electric field stimulation (EFS) using bipolar platinum electrodes approximately 1 cm apart, oriented along the length of the strip using a stimulus of 12 volts with a 1 ms pulse duration. EFS was induced using a Grass S88 stimulator (Natus Neurology Inc., Warwich, RI) interfaced with a Stimu-Splitter II (Med-Lab Instruments, Loveland, CO) power amplifier. A frequency response curve was constructed for each strip (2, 5, 12, 20, and 30 Hz). The strips were allowed to equilibrate 2 minutes before the next stimulation. Following this frequency response, the strips were exposed to either 1 µM atropine or 30 µM of α,β-methylene ATP for 30 minutes and the stimulation was repeated. After the second frequency response, the strips that were previously exposed to atropine were treated with α,β-methylene ATP, and the strips that were previously exposed to α,β-methylene ATP were treated with atropine for 30 minutes. A third frequency response was performed after the last treatments. Preliminary experiments showed no differences between 3 successive frequency response curves in the absence of atropine or α,β-methylene ATP (data not shown).

### Histological analysis

Pelvic organs from 3 age-matched control mice and 3 CIE mice were harvested and formalin-fixed, taking care to ensure that the bladder was empty. The bladder with associated genitourinary and pelvic floor tissues were collected *en bloc* (i.e. bladder and attached genitourinary structures and muscles were carefully separated from vertebrae and os coxae, and sectioned anterior-posterior as a block of tissue), equilibrated in 30% sucrose with phosphate buffer for 3 days, and frozen for cryo-sectioning. A series of adjacent sections were cut on a cryostat. Each section was 12 µm thick, and mounted onto charged slides (Superfrost Plus slides, Fisherbrand, #12-550-15). After drying, the sections were stained with hematoxylin and eosin (H&E), dehydrated and cover slipped with DPX mounting medium. The thickness of the bladder layers (urothelium, mucosa, and detrusor muscle), was measured at 40x magnification using BioQuant software (Nashville, TN). For this, three sections per bladder, separated by at least 40 µm, were analyzed. The inner and outer margin of each layer was traced with the Auto-Width tool of the BioQuant Image Analysis program, and the average thickness of each layer automatically generated at 10 µm intervals. A total of 91±8 measurements were made per layer and per bladder, and the results averaged for each layer and bladder.

### Statistics

All data is presented as mean ± SEM. For individual comparisons, statistical differences were determined by either Student’s t-test or a nonparametric statistic (Wilcoxon Rank Sum/Mann-Whitney U-test) when the variance between groups was non-homogenous. To determine statistical differences between multiple groups, analysis of variance with Newman–Keuls *post-hoc* comparisons was performed. However, if the variance between groups was non-homogenous, a non-parametric statistic (Wilcoxon Rank Sum/Mann-Whitney U-test) was used.

## Results

### Effect of CIE on mice

**Figure 2:**
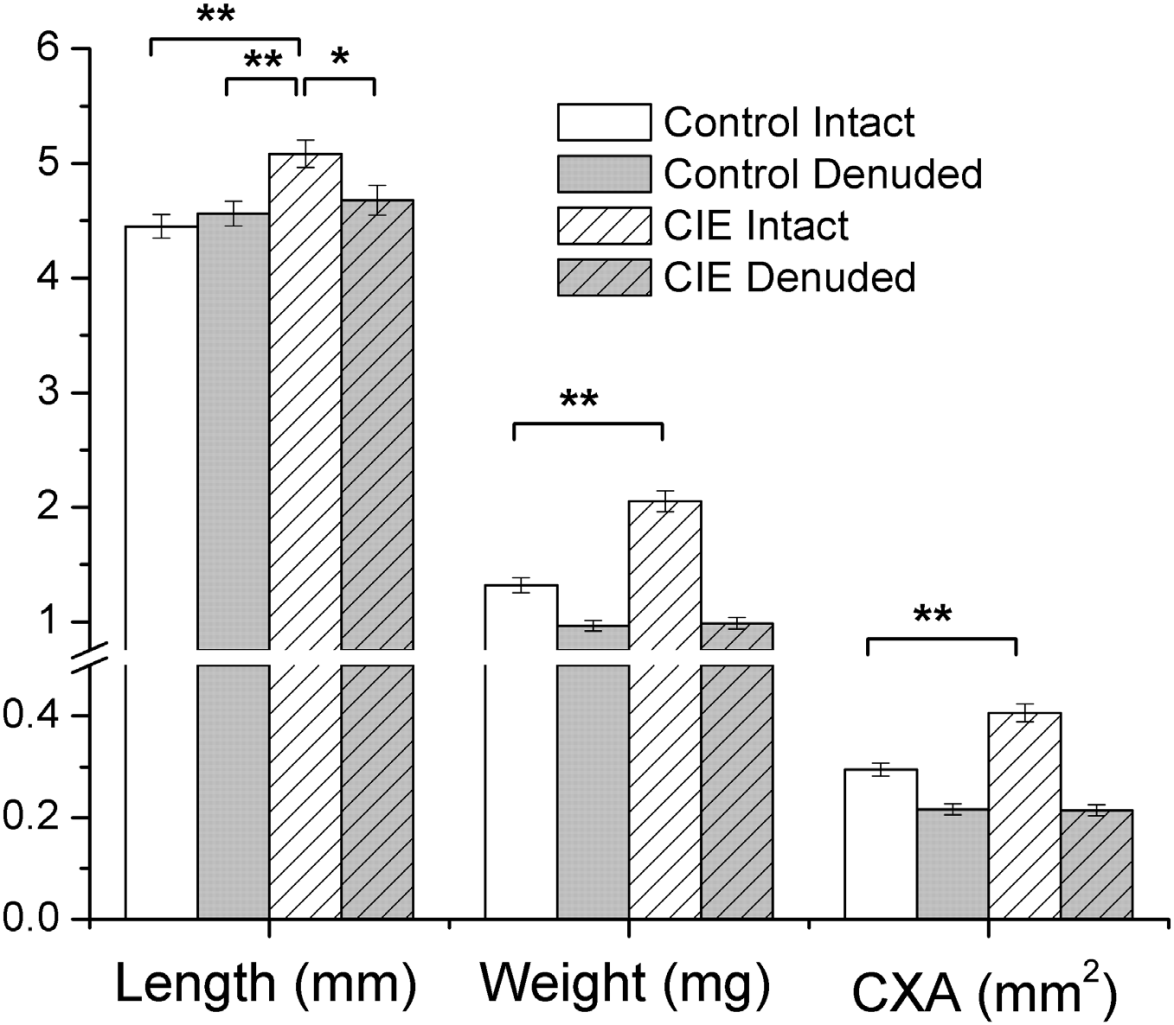
Measurements of individual bladder strips following tension experiments. Significant differences are denoted as follows: *denotes p<0.05; **denotes p<0.01.

**Figure 3:**
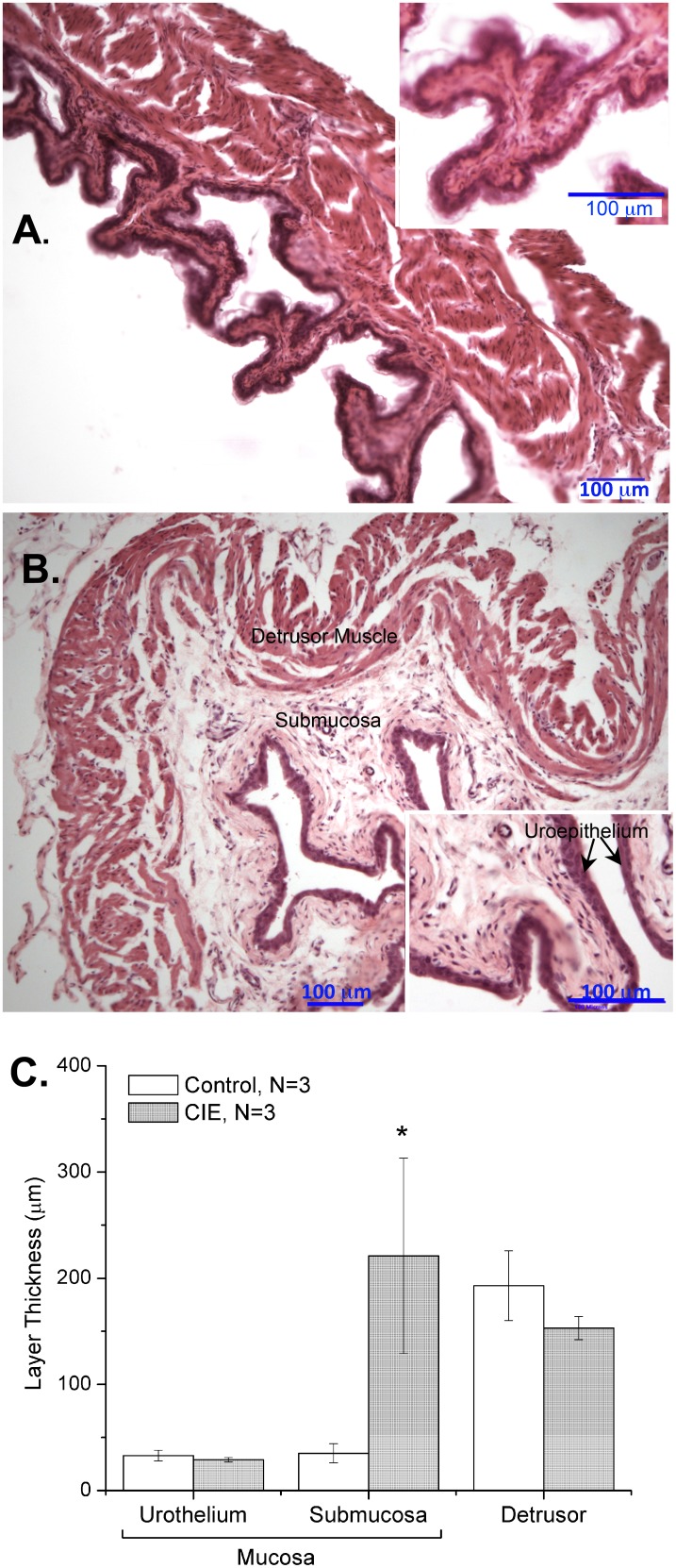
Bladder histology and layer thickness. 40x micrograph (H&E images) showing thickness of three layers of the bladder wall in age matched control mouse bladder (A) and CIE mouse bladder (B). Insets showing detail of urothelial layer. (C) Quantification of layer thickness using BioQuant image analysis software. Average layer thickness was compared between mice, such that N reflected the number of mice. *denotes significance at p<0.05.

At 4 weeks post-inoculation, the average CSS for CIE mice is approximately 1 [14], [15]. The average maximum CSS for the mice used in this study was 2.0±0.12 (range 1–5). Most mice had a CSS maximum of 1 or 2. There were no statistically significant correlations between CSS score and any outcome measure in this study including: the potassium-induced contraction, nerve-mediated maximal contraction, bethanechol-induced maximal contraction or bethanechol potency, the purinergic-induced maximal contraction, the potency of α,β-methylene ATP, or the percent of the purinergic and cholinergic components of nerve mediated contraction. Mice with CIE (N = 54) weighed significantly less (p<0.01) than controls (N = 40, 24.9±0.3 and 26.8±0.3 g, respectively), whereas the weight of bladders from mice with CIE was not different in comparison to the bladders from control mice (23.5±1 mg and 23.1±0.5 mg, respectively). Subsequent to the *in* *vitro* contractility studies, the length and weight of each bladder strip was measured to determine cross-sectional area (CXA; Figure 2). There was no difference in the CXA between mucosa denuded CIE (0.21±0.01 mm^2^) and mucosa denuded control bladder strips (0.21±0.01 mm^2^). However, in mucosa intact bladder strips, the CXA was significantly (p<0.01) greater in bladder strips from CIE (0.41±0.02 mm^2^) compared to control mice (0.29±0.01 mm^2^). This suggests there is mucosal hypertrophy, but not muscle hypertrophy in CIE mice bladders. Histological examination of the tissues (Figure 3) confirmed a significant increase in the thickness of the submucosal layer in CIE bladders (221.4±92 µm) compared to control bladders (35.3±9.0 µm). The urothelial layer in CIE bladders (28.6±2.4 µm) was not different from control bladders (33.19±5.1 µm). In addition, the thickness of the detrusor smooth muscle layer was similar in CIE and controls (153.3±11 and 192.6±33 µm, respectively). Staining for myelin basic protein was observed within the bladder wall and pudendal nerve of CIE mice, showing similar patterns to age matched control mice (Figure 1), thus no evidence of peripheral demyelination was observed in this model.

### Effect of CIE on potassium-induced contractions

**Figure 4:**
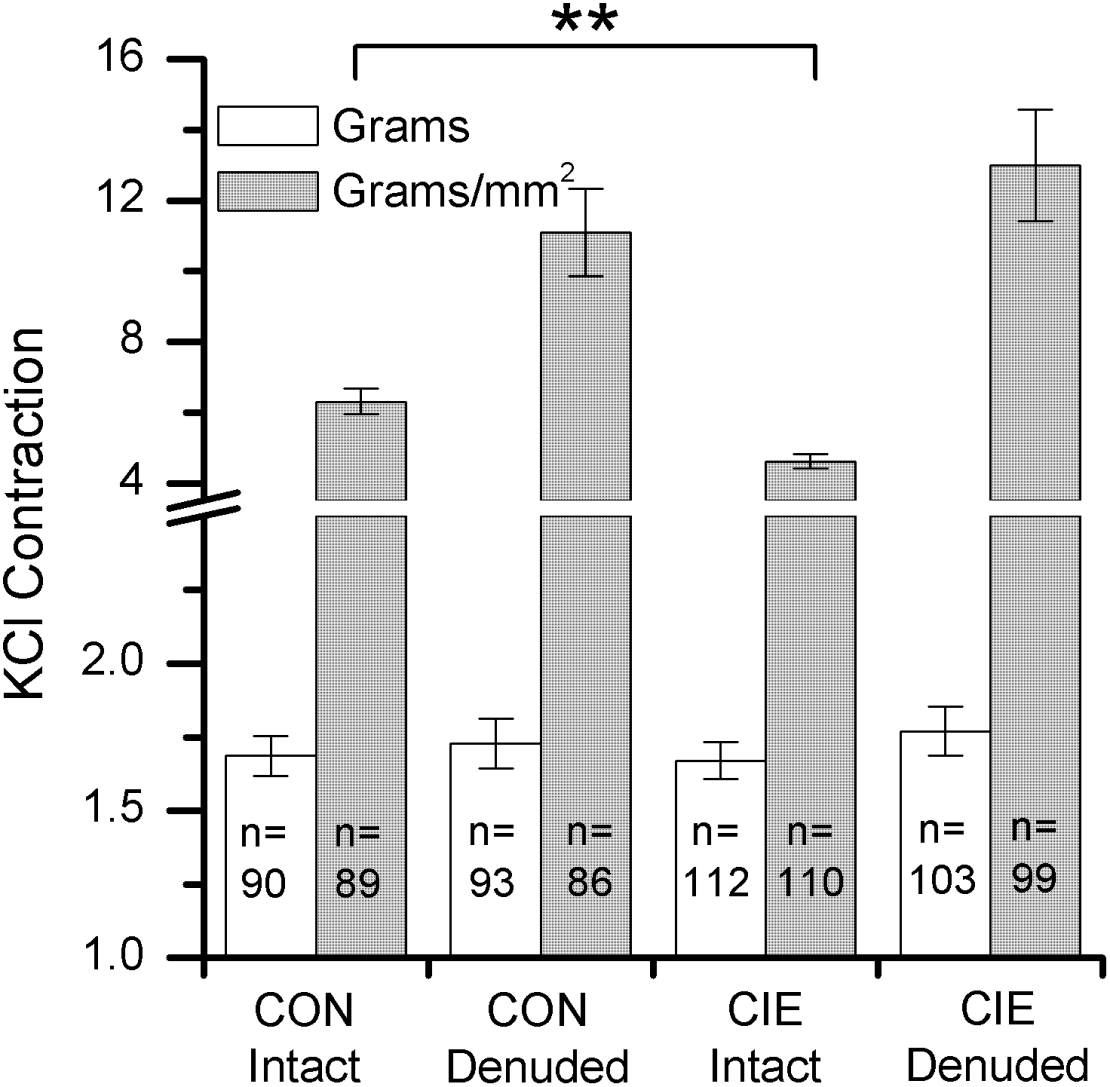
Potassium-induced contraction in mouse bladder strips. Mucosa intact and mucosa denuded bladder strips were exposed to high potassium buffer to induce contraction. **denotes significant (p<0.01) differences. The data is presented in terms of both grams and grams per cross sectional area (g/mm^2^). CON denotes age-matched control.

Prior to all agonist- or nerve-induced contraction studies, the bladder strips were induced to contract with an isotonic buffer containing 120 mM potassium. There was no difference in the potassium-induced maximal contraction between mucosa denuded bladder strips from CIE and control mice either in terms of grams or grams/mm^2^ (Figure 4). This suggests that CIE does not induce a deficit in the ability of the muscle to generate force. In contrast, even though the maximal potassium-induced contraction in terms of grams in mucosa intact bladder strips from CIE and control mice were not different, the potassium-induced contraction was significantly (p<0.01) less in bladder strips from CIE mice compared to controls when normalized to CXA. This reduction in potassium contraction in intact CIE mouse bladder strips may be the result of the mucosal hypertrophy and, hence, an increased CXA in mucosa intact bladders from CIE mice.

### Effect of CIE on bethanechol-induced contraction

**Figure 5:**
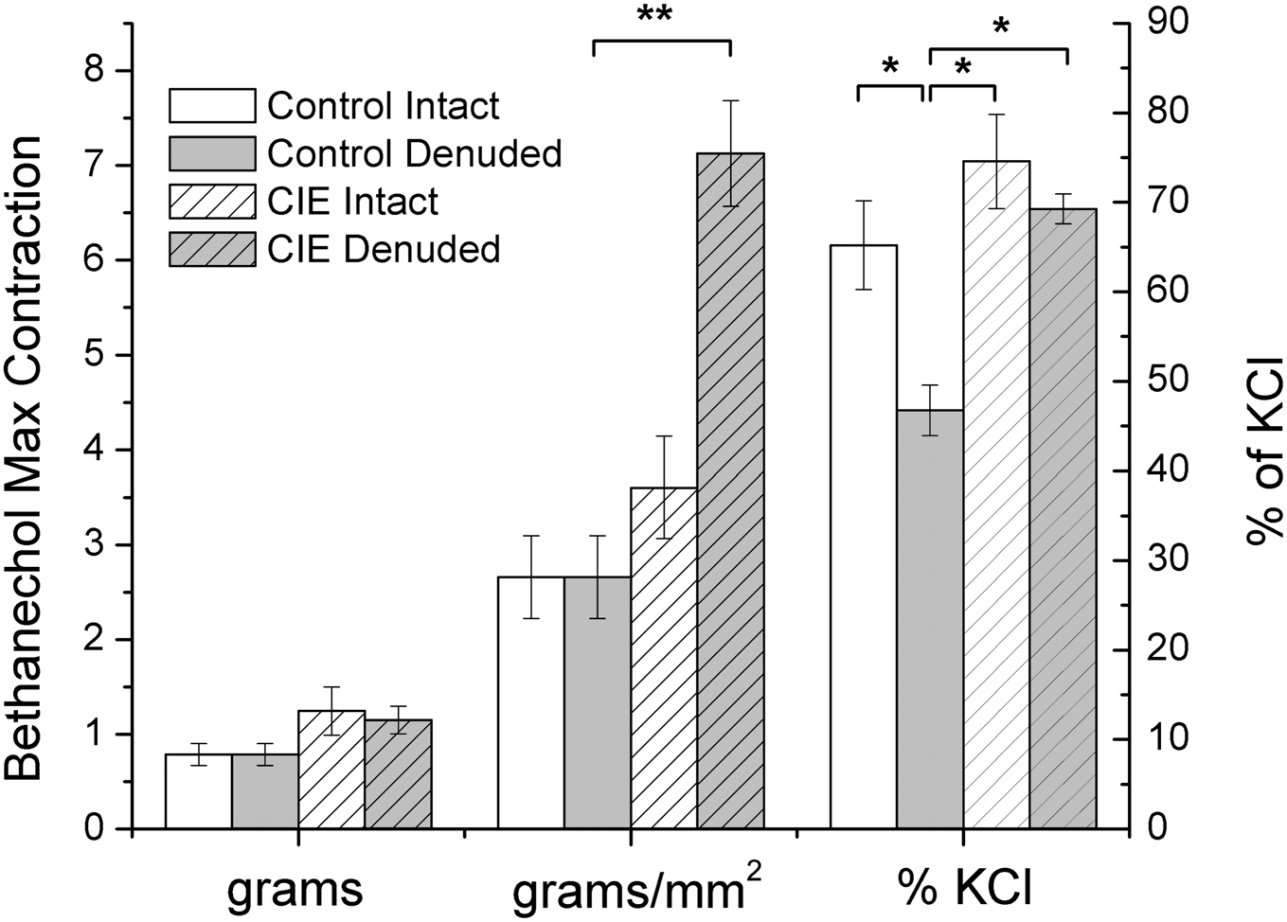
Bethanechol-induced contraction in mouse bladder strips. Mucosa intact and mucosa denuded bladder strips were subjected to a cumulative bethanechol concentration response curve at half log intervals from 1E-7 M to 1E-2 M. The maximal contraction is shown in terms of grams, grams/mm^2^, and percent of high potassium-induced contraction (% KCl). Significant differences are denoted as follows: *p<0.05; **p<0.01.

The detrusor smooth muscle of CIE mice responded greater to exogenous bethanechol than age matched control mice. Figure 5 shows a two-fold greater contraction in CIE denuded *vs* age matched control denuded strips, normalized to CXA (grams/mm^2^). CXA of these groups was nearly identical (Figure 2), and does not account for the observed difference. In age matched control mice, the maximal bethanechol-induced contraction (% KCl) was significantly less in mucosa denuded compared to mucosa intact bladder strips. This suggests that the mucosa augments the cholinergic-induced maximal contraction in control, an effect not seen in CIE mouse bladders. The potency of bethanechol (Table 1) was significantly increased (lower EC_50_) in CIE bladder strips *vs* age matched controls (both mucosa intact and mucosa denuded). Removal of the mucosa from control or CIE mouse bladders had no effect on the potency of bethanechol to induce contraction.

**Table 1:** Bethanechol maximal contraction and potency and the potency of subtype selective muscarinic receptor antagonists for inhibition of bethanechol induced contraction.

	BETH MAX(% KCl)	BETH EC_50_ (µM)	DAR pKb (95% CI)	METH pKb (95% CI)
CON intact	65±5.0	19±2.0^b^	8.8 (8.6–9.0)	6.1 (5.9–6.3)
CON denuded	47±2.8^a^	19±2.0^b^^,^ c	9.4 (9.1–9.6)	5.9 (5.6–6.2)
CIE intact	75±5.2	10±2.0	9.2 (9.0–9.4)	6.4 (6.0–6.8)
CIE denuded	69±1.7	14±1.5	9.9 (9.5–10.1)	6.1 (5.9–6.3)

Potency values were determined by Schild analysis for the M_3_ selective antagonist darifenacin and the M_2_ selective antagonist methoctramine. 3 concentrations of each antagonist were used to generate the Schild plots.

a denotes significant difference (p<0.05) between aged matched control intact and CIE intact and denuded.

b denotes significant difference (p<0.05) from CIE intact.

c denotes significant difference (p<0.05) from CIE denuded.

### Muscarinic receptor antagonist potency for inhibition of bethanechol-induced contraction

As can be seen in Table 1, the M_3_ subtype selective antagonist darifenacin was highly potent for inhibition of bethanechol-induced contraction in both mucosa intact CIE and control bladder strips (pKb = 9.2 and 8.8 respectively, overlapping 95% confidence interval) consistent with the M_3_ muscarinic receptor mediating bladder contraction. Removal of the mucosa significantly (non-overlapping 95% confidence intervals) increased darifenacin potency in both CIE and control bladder strips (pKb = 9.9 and 9.4, respectively). The M_2_ selective antagonist methoctramine had low potency for inhibition of bethanechol-induced contraction of mucosa intact and mucosa denuded CIE and control bladders (Table 1), also consistent with the M_3_ muscarinic receptor mediating cholinergic-induced contraction in both CIE and age-matched control bladders.

### Purinergic concentration response curves

**Figure 6:**
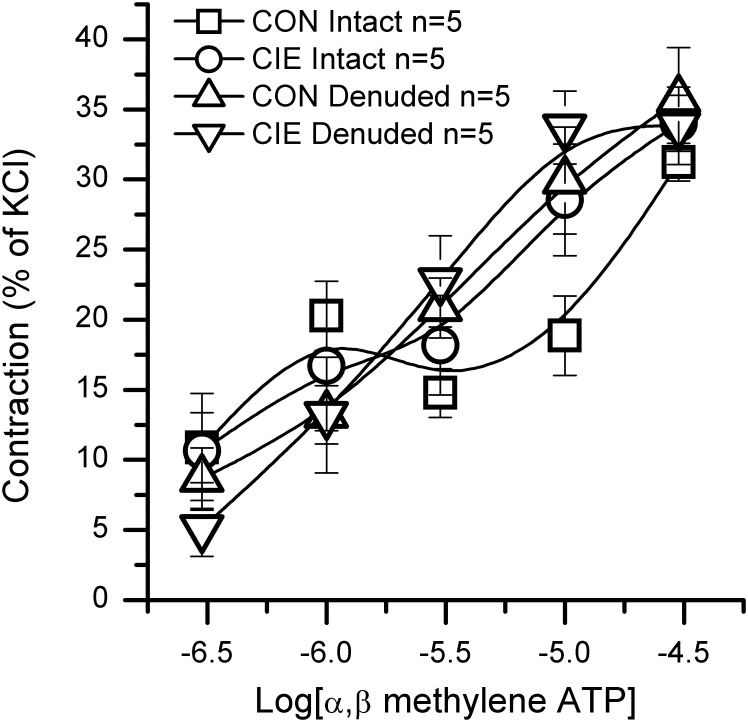
α,β-methylene ATP concentration response curves (CRCs) in mucosa intact and mucosa denuded bladder strips. Non-cumulative α,β-methylene ATP CRCs from age-matched control (CON) and CIE mouse bladder strips are presented as the percent of the high potassium-induced contraction (% KCl).

Concentration response curves to the P2X receptor agonist α,β-methylene ATP were generated by the non-cumulative addition of agonist for CIE and control bladder strips, both mucosa denuded and mucosa intact. There were no differences in the maximal contraction to α,β-methylene ATP between control and CIE bladder strips, either mucosa denuded or mucosa intact. In addition, the EC_50_ values were similar for all groups, ranging from 1.4 to 1.9 µM (Figure 6).

### Nerve-evoked contractions

**Figure 7:**
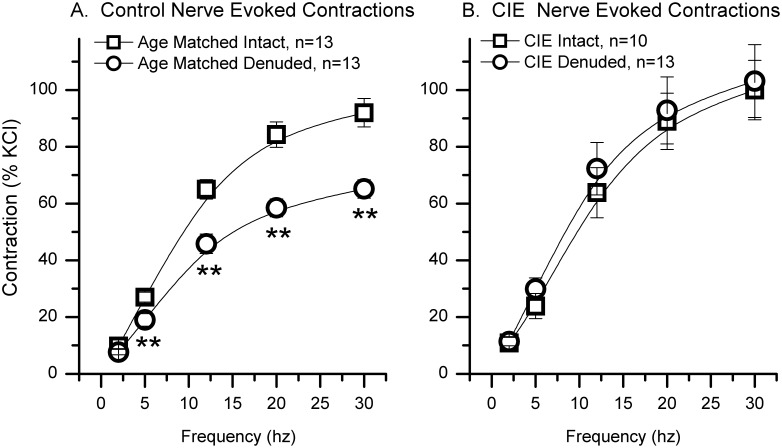
Mucosal influence on nerve-evoked contractions in age-matched control and CIE bladder strips. EFS (12 V, 1 ms pulse duration) was used to induce nerve-evoked contractions in aged-matched control (A) and CIE (B) mouse bladder strips. The data is presented as the percent of the high potassium-induced contraction. **denotes significant (p<0.01) differences between mucosa intact and mucosa denuded.

Nerve-evoked contractions were induced by EFS using trains of pulses of 12 V, 1 ms pulse duration with varying frequencies (2, 5, 12, 20 and 30 Hz). EFS-induced contractions were virtually eliminated (>95%) in the presence of 1 µM of tetrodotoxin (TTX, a blocker of nerve transmission in nerve fibers), confirming that these contractions were nerve-evoked (data not shown). There was no difference in the maximal nerve-evoked contraction between mucosa intact bladder strips from control and CIE mice (Figure 7, A and B). Removal of the mucosa had no effect on the maximal nerve-evoked contraction in bladder strips from CIE mice, however, removal of the mucosa from control bladder strips significantly reduced the maximal nerve-evoked contraction at frequencies of 5 Hz and above. This suggests that the mucosa augments nerve-evoked contraction in control but not in CIE bladders, possibly, through nerve-mediated release of either acetylcholine (ACh) or ATP from the mucosa.

### Effect of cholinergic and purinergic blockade on EFS-induced contraction

**Figure 8:**
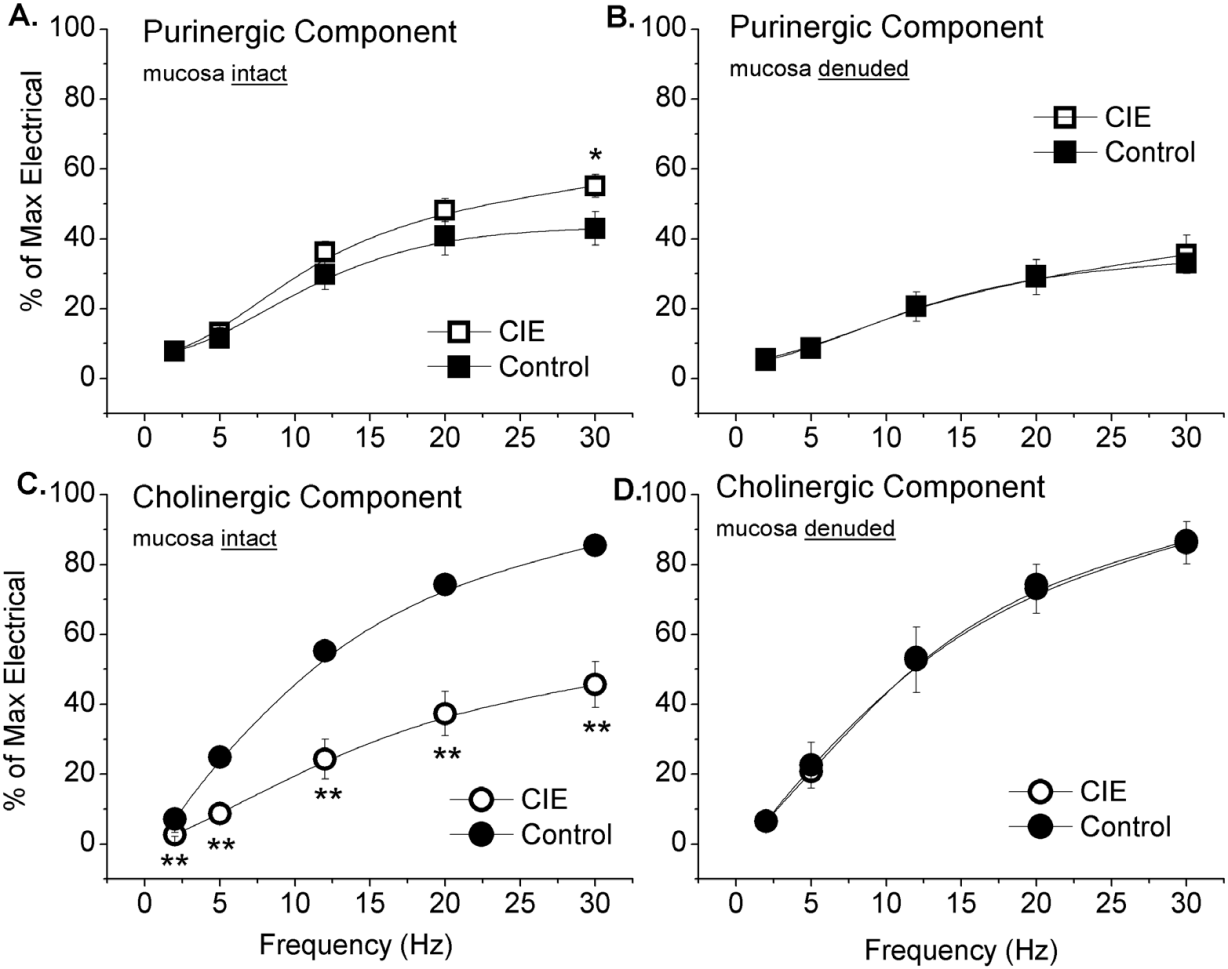
Cholinergic and purinergic components of nerve-evoked contraction in CIE and age matched control mouse bladders. EFS (12 V, 1 ms pulse duration) was used to induce nerve-evoked contractions in aged-matched control and CIE mouse bladder strips. The purinergic component (A and B) was determined in the presence of 1 µM atropine. The cholinergic component (C and D) was determined following purinergic desensitization with 30 µM α,β-methylene ATP. Data is presented as the percent of the maximal EFS-induced contraction prior to either cholinergic or purinergic inhibition. Significant differences are denoted as follows: *denotes p<0.05; **denotes p<0.01.

In mucosa intact bladder strips isolated from control mice, the non-selective muscarinic receptor antagonist atropine (1 µM) reduced the nerve-evoked maximal contraction by 57±5% (p<0.01; Figure 8A, solid squares). To a lesser degree, desensitization of purinergic receptors (P2X) with 30 µM α,β-methylene ATP also reduced the nerve-evoked maximal contraction by 15±3% (p<0.01; Figure 8C, solid circles). The combination of both drugs reduced nerve-evoked maximal contractions by 96±0.6% (p<0.01; not shown), confirming these two neurotransmitters are responsible for nearly all of the nerve-evoked contraction. Removal of the mucosa had no significant impact on the inhibitory effects of atropine, α,β-methylene ATP (Figure 8, A, B, C, and D) or the combination of both.

The cholinergic and purinergic components of nerve-evoked contractions were quite different in mucosa intact CIE mouse bladder strips. Purinergic stimulation (Figure 8A, open squares) mediated 55±3% while cholinergic stimulation (Figure 8C, open circles) mediated 46±7% of the maximal nerve-evoked contractions. Thus, the cholinergic component of nerve-evoked contractions was reduced (p<0.01) in CIE mouse bladders, while the purinergic component was increased (p<0.05) in CIE mouse bladders, compared to controls.

These alterations in the purinergic and cholinergic components to nerve-evoked muscle contractions were mediated by the mucosa in CIE mice. Removal of the mucosa in CIE mouse bladders increased (p<0.01) the cholinergic component of nerve-evoked contraction to 87±1% (Figure 8D, open circles) and decreased (p<0.01) the purinergic component to 35±6% (Figure 8B, open squares). In mucosa denuded CIE mouse bladders, the relative magnitude of the cholinergic and purinergic components of nerve-evoked contraction were not different from controls (Figure 8, B and D).

## Discussion

CIE in mice leads to neurogenic detrusor overactivity, which has recently been characterized [14], [15]. Cystometry recordings in awake, unrestrained animals revealed that CIE mice have shortened inter-micturition intervals, lower voided volumes, and an elevated number of non-voiding contractions in comparison with controls. Although gross histological analysis of bladders from CIE and control mice showed no substantial differences in our previous report [14], detailed quantitative histological analysis of the bladders from CIE mice presented here demonstrated organ-level hypertrophy that appeared to be a result of edema, primarily in the submucosal layer. No granulocytes were observed, and few lymphocytes, suggesting the edema may be related to increased permeability rather than inflammation. This idea is supported by a previous study demonstrating increased urothelial permeability within several hours of spinal cord injury in rats [18]. This study also provides solid evidence of a connection between the nervous system and the urothelium, which is supported by our findings as well.

We examined changes in motor innervation and contractile response to exogenous agonists in CIE mice, while also determining the role of the mucosa in the observed changes. ACh and ATP are both released from terminals of parasympathetic nerves during micturition; however the relative contribution of each cotransmitter is known to vary by species and disease state [19]. The influence of the mucosa on bladder contraction also varies by species. For example, in pig bladders the mucosa suppresses contraction [20], whereas in normal mice the mucosa potentiates contractile responses, both to bethanechol and electric field stimulation (nerve-evoked contractions). There is a role for an “atropine resistant” (non-cholinergic) component of normal bladder contraction in many mammals, excluding man [21]. In normal mice, a purinergic component of nerve-evoked contractions was observed, reaching a magnitude of approximately 40% of the maximum nerve-evoked contraction without atropine. The removal of the mucosa did not alter the relative magnitude of either the purinergic or cholinergic components of nerve-evoked contractions (Figure 8), but did decrease their absolute magnitude. However, in CIE mice, the mucosa no longer enhances the magnitude of the nerve-evoked or bethanechol-induced contraction. Instead, mucosa from CIE mouse bladder decreases the cholinergic component of nerve-evoked contraction, while augmenting the purinergic component. This is consistent with an increased role for ATP observed in various human bladder pathologies, such as outlet obstruction due to enlarged prostate [22], overactive bladder, neurogenic bladder, and interstitial cystitis [23]. As previously mentioned, human bladder strips do not show atropine-resistant contractions in the absence of pathology [22], [23].

Removal of the mucosa from CIE mouse bladders resulted in nerve-evoked contractions, which were similar to mucosa intact control bladders. The augmentation of nerve-evoked and bethanechol-induced contractions observed in mucosa intact age matched controls is lost in CIE bladders. Rather, the presence of the mucosa in intact CIE bladders decreased the relative magnitude of the cholinergic component and enhanced the purinergic component of nerve-evoked contractions without altering the magnitude of the contractile response. Therefore, it is evident that the relative contribution of the two neurotransmitters is influenced by the presence of the mucosa. This may be, in part, due to altered release of various substances influencing smooth muscle contraction, or perhaps to alterations in the interstitial cells relaying mucosal signals to smooth muscle of the detrusor.

The urothelium is known to release various substances, such as ACh, ATP, nitric oxide [24], prostaglandins, and nerve growth factor [25], [26]. These substances could have either inhibitory or augmenting effects on muscarinic receptor-mediated detrusor contractions. There is also evidence for a mucosal-derived, yet unidentified, factor that inhibits bladder contraction [27], [28]. Spermidine is an endogenous polycationic amine which acts in a receptor-independent fashion, by modulating ion channels, including calcium channels [29]. Increased spermidine production and release has been shown in cultured urothelial cells from patients with overactive bladder [26]. Polyamines including spermidine also have been shown to inhibit ACh-induced contraction of gastrointestinal smooth muscle [30] and trigger relaxation of ACh-induced contractions in the rat bladder [29]. Whether there is an alteration of urothelial spermidine production and release in mice with bladder overactivity, such as seen in CIE, is still unknown.

Our data suggests that mucosal dysfunction developed in CIE mouse bladders. This dysfunction may be related to the altered urothelial release of mediators of contraction described above, and/or altered permeability which is supported by the edema found in the submucosal layer, creating organ-level hypertrophy. Additionally, changes in expression levels of several receptors have been shown in EAE mice (a different model of MS), including an up-regulation of M_2_ and P2X_3_ receptor transcripts in the mucosa and detrusor smooth muscle [31]. As we have previously shown, in various bladder pathologies, there is an increased role of the M_2_ subtype in mediating contraction [32]. However, our subtype-selective antagonist data support the role of the M_3_ receptor predominantly mediating the contraction. Also, we have previously shown that M_3_ transcript density is not correlated with protein levels across various rat bladder pathologies [33].

In both control and CIE bladders, the potency of the M_3_ selective antagonist darifenacin was increased by removal of the mucosa. This may suggest that mucosal M_2_ receptors participate in the bethanechol-induced contraction of these strips. Urothelial M_2_ receptors have been shown to mediate the release of ATP and PGE_2_ and both these compounds can stimulate ACh release from the urothelium [33]. The urothelial released ACh may act on muscarinic receptors and the urothelial released ATP may act on purinergic receptors to enhance contraction. Additional ATP and ACh released in response to bethanechol activation of mucosal M_2_ receptors would tend to reduce the darifenacin-induced shift in the bethanechol dose response curve, but would not reduce the methoctramine-induced shift. Because darifenacin potency is increased to a similar degree upon removal of the mucosa in both CIE and control bladder strips, CIE may not alter this urothelial modulatory mechanism.

No hypertrophy was observed in the detrusor smooth muscle of CIE mice, however we did note a two-fold increase in the maximum response to the non-selective muscarinic agonist bethanechol in terms of normalized force (g/mm^2^). This may result from up-regulation of muscarinic receptors in the detrusor smooth muscle of CIE mice, which is further supported by an increase in bethanechol potency in CIE bladder, as well as an increase in the bethanechol-induced maximum contractions (% KCl in denuded CIE vs control). This might be similar to the phenomenon of muscarinic supersensitivity following outlet obstruction [34]. Accumulation of lesions in the CNS as a result of CIE in mice, or MS in humans, will eventually disrupt or alter the complex neuronal control over micturition. This CIE mouse model shows demyelination primarily in the CNS; no demyelination in peripheral nerves was observed in the bladder wall. Yet, a distinct phenotype of neurogenic bladder dysfunction is observed, characterized by an increased number of non-voiding contractions and shortened inter-micturition interval. These symptoms are similar to those observed in upper spinal cord injuries, which suggests that the interruptions caused by MS-induced lesions result in decreased central control, resembling spinal cord injury. These cystometry findings are thought to be a function of increased afferent activity, suggesting a loss of descending inhibition from the pontine micturition center. Our data suggests there are changes in the motor (efferent) innervation as well, and that the mucosa plays a significant role in modulation of efferent signals. Previous studies have shown a tight link between the urothelium and nervous system [18], and a number of alterations in urothelial cells under pathological conditions. While our data supports this idea, further studies are required to definitively conclude which cell type(s) are altered in these mice, and to examine changes to bladder afferents.

## Conclusions

In summary, our data demonstrate a deficit in the nerve-evoked cholinergic component of contraction in CIE mice, which is not due to the ability of the smooth muscle to respond to acetylcholine. In fact, bethanechol potency and maximal contractile response were increased. We conclude that neurodegenerative bladder dysfunction in this model of multiple sclerosis may be, in part, due to pathologic changes in the urothelium and bladder mucosa that caused suppression of muscarinic receptor-mediated contractile response and augmentation of the purinergic response of the underlying smooth muscle.
